# Actinomycetes Associated with Arthropods as a Source of New Bioactive Compounds

**DOI:** 10.3390/cimb46050238

**Published:** 2024-04-24

**Authors:** Carlos Olano, Miriam Rodríguez

**Affiliations:** 1 Departamento de Biología Funcional e Instituto Universitario de Oncología del Principado de Asturias (IUOPA), Universidad de Oviedo, 33006 Oviedo, Spain; olanocarlos@uniovi.es; 2 Instituto de Investigación Sanitaria del Principado de Asturias (ISPA), 33011 Oviedo, Spain

**Keywords:** actinomycetes, bioactive compounds, arthropods symbionts, drug discovery, natural products

## Abstract

Antimicrobial resistance is one of the main global threats to human health in the 21st century due to the rapid appearance of bacterial resistance and the lack of novel bioactive compounds. Natural products, especially from Actinomycetes, remain the best source to refill the drug industry pipeline. Different strategies have been pursued to increase the chances of discovering new molecules, such as studying underexplored environments like arthropod symbionts, which represent a relevant reservoir for active metabolites. This review summarizes recent research on the identification of bioactive molecules produced by Actinomycetes associated with arthropods’ microbiome. The metabolites have been categorized based on their structural properties and host, highlighting that multidisciplinary approaches will be the key to fully understanding this complex relationship.

## 1. Introduction

In recent times, healthcare systems across the globe have faced unprecedented challenges due to the emergence of new infectious diseases and pathogens, as well as a sharp increase in antibiotic-resistant bacteria. Moreover, the recent pandemic caused by the SARS-CoV-2 virus serves as a stark reminder of this reality. The pandemic has negatively impacted antibiotic stewardship, resulting in increased global usage of antibiotics, which has exacerbated the problem of antibiotic resistance [1]. In 2019 alone, it is estimated that bacterial antimicrobial resistance was directly responsible for 1.27 million global deaths and contributed to 4.95 million deceases, and this is projected to rise to around 10 million deaths annually by 2050 if insufficient measures are taken [2,3]. These alarming data highlighted the urgent need for finding new compounds with novel bioactivities.

Despite the rising popularity of synthetic drug design, natural products (NPs) continue to be one of the best sources for developing new drugs because NPs are unique in their chemical diversity and effectiveness as antibiotics [1]. According to Newman and Cragg [4], drugs obtained from nature account for approximately 23% of 1881 new approved drugs discovered between January 1981 and September 2019. Furthermore, only in 2023, the U.S. FDA (Food and Drug Administration) approved ten new NPs for clinical use [5]. NPs have been discovered in a wide variety of organisms, including plants, vertebrates, and invertebrates [6]. However, most of the bioactive compounds have been isolated from microbes, especially by the phylum *Actinomycetota*, formally known as *Actinobacteria* [7]. This group includes the Class *Actinomycetia*, commonly referred to as Actinomycetes, which is a ubiquitous filamentous bacterium, Gram-positive with a GC-rich linear genome [8]. Actinomycetes are found in different habitats, demonstrating a unique versatility and adaptability to varying environments [9]. They are responsible for approximately two-thirds of all naturally derived antibiotics, with members of the genus *Streptomyces* accounting for up to 80% of the antimicrobials used in clinical care [10]. They also produce an array of bioactive metabolites such as antitumoral, antifungal, antiparasitic, biopesticidal, and antioxidant, among others [6]. In addition, rare Actinomycetes such as *Micromonospora*, *Nocardiopsis*, *Pseudonocardia*, *Nocardia*, *Amycolatopsis*, and *Actinomadura* have gained relevance in recent years and have accounted for the remaining actinomycete-derived antibiotics [11].

Regardless of the significant role that Actinomycetes play in drug discovery, there has been a decline in the identification of new NPs in recent decades. Nowadays, the major drawback is the re-isolation of compounds that are already known. During the golden age of antibiotic discovery (from 1940 to 1970), novel classes of antibiotics were being discovered on an almost yearly basis by isolation from soil samples. This led to the discovery of a large number of novel NPs such as tetracyclines or vancomycin. Unfortunately, this success had as a secondary consequence, being the depletion of the traditional bioactive metabolite sources [7]. To solve this problem and increase the likelihood of discovering new active molecules innovative research methodologies have been employed [12,13,14]. Genome mining, for instance, emerged in the early 2000s thanks to advances in genome sequencing, bioinformatics tools, and the understanding of secondary metabolite biosynthesis. Surprisingly, genome mining studies have demonstrated that Actinomycetes, such as *Streptomyces*, possess between 25 and 70 biosynthetic gene clusters (BGCs). This is much more than initially believed, and it shows that only a small fraction of these bioactive compounds are synthesized in laboratory settings using traditional culture methods [15,16]. The last issue highlights the fact that the majority of biodiversity remains untapped for the discovery of novel NPs [17]. Although genome mining has opened up new opportunities for identifying in silico new BGC, it also requires extensive laboratory work, since BGCs are sometimes silent or low-expressed, and the products are not always observed under laboratory conditions [18]. Another relevant methodology to overcome these problems is combinatorial biosynthesis, which uses different genetic engineering approaches to modify biosynthetic pathways to generate new products with different or improved properties using nature’s biosynthetic machinery [19]. On the other hand, we cannot ignore the fact that, recently, the discovery of novel antibiotic-producing strains and new bioactive compounds in under-explored or unexplored environments has revitalized the NPs field [20]. Actinomycetes, for example, have been found in unusual environments, including symbiotic associations with plants, fungi, vertebrates, or invertebrate animals, both marine and terrestrial. One of the most widespread examples is the symbiotic relationship formed with arthropods, especially with insects [21,22,23].

The present review aims to deliver an in-depth analysis of the latest research on NPs discovered from Actinomycetes that form symbiotic relationships with arthropods. The focus is on categorizing these compounds by their structural properties and host. Finally, it emphasizes the immense potential of Actinomycetes linked with arthropods, especially insects, as an invaluable source of bioactive compounds.

To conduct this work, we searched and selected sources consulting Web of Science, Google Scholar, and PubMed. We used keywords such as arthropods, symbionts, insects, actinomycetes, bioactive compounds, and natural products. Our inclusion criteria were that the research was recent, the compounds described were produced by actinomycetes isolated from arthropods, and were bioactive metabolites.

## 2. Actinomycetes and Arthropods Relationship

The phylum of arthropods is the most abundant and varied in the animal kingdom. This group includes invertebrate animals with an external skeleton and articulated appendages such as insects, arachnids, crustaceans, and myriapods, which make up about 50% of the Earth’s animal biomass [24]. Arthropods have demonstrated remarkable adaptability in thriving across diverse environments, including marine, freshwater, terrestrial, and aerial ecosystems. Sometimes, microorganisms and arthropods have developed mutually beneficial symbiosis relationships through close interaction and signal exchange. In that sense, NPs play a vital role in functions like defence, protection, behaviour, and virulence, as well as central physiological processes [25]. For example, they can act as signalling molecules that can regulate the individual development and reproduction of chemical mediators between natural enemies and hosts, acting as defence agents against predators across different species [23]. Protective symbiosis is most prevalent in arthropods, and it has different purposes for safeguarding the insect’s nutritional resources, such as fungus-growing termites and ants [26], or providing self-protection like solitary wasps that use *Streptomyces* bacteria to protect their pupae in brood chambers [27]. It was not surprising that the order *Actinomycetales*, particularly the genus *Streptomyces*, has the most known antimicrobialsactive symbionts associated with arthropods [21].

Actinomycetes have established a close and long-lasting bond with arthropods and can inhabit various parts of their body such as the gut or the external surface, or inhabit their habitat, such as their food stores or nests. Screening research has primarily focused on specific regions, mainly central America, southern Brazil, China, South Africa, and South Korea. Traditionally, the identification of symbiotic actinomycetes has been done through arthropod collection, sample processing, and microbial isolation. The classical isolation method was commonly used in many screenings, often with a preference for isolating *Streptomyces* over other species. However, newer techniques have emerged, such as metagenome studies of microbial communities, which can provide valuable information like identifying strains that are difficult to cultivate under laboratory conditions or finding silent or low-expressed BGCs. The next step in the process is usually antimicrobial screening, which is crucial for identifying interesting symbionts. Finally, the active compounds are fermented and isolated. It is worth noting that only in some cases were the activity and ecology of isolated compounds not commonly studied together [23].

Currently, actinomycetes symbionts are mainly isolated from insects, which are approximately one-sixth of a part of the described arthropods [28]. Insects are an incredibly diverse and widespread group of animals that play a crucial role in ecosystems like pollination, plant biomass consumption, and the spread of diseases. The most extensively studied insects in this regard have been ants, termites, bees, beetles, and grasshoppers. Interestingly, Actinomycetes, particularly *Streptomyces* strains associated with insects, have been reported to have higher antimicrobial activity than those found dwelling on plant material or soil [22,23].

The use of arthropods for medicinal purposes is not something new; it has been practiced for centuries across numerous countries around the globe, for example, the application of maggots of *Lucilia sericata* for wound healing has been well documented [29]. Despite this, the potential to utilize these bioactivities as an innovative alternative to conventional pharmaceuticals for disease treatment remains largely unexplored [30]. This could be due to some challenges that exist in the clinical application of NPs, such as their limited solubility, inappropriate molecular size, or instability, among others [31]. Nevertheless, there has been a consistent increase in research covering actinomycetes associated with arthropods as a source of new bioactive compounds [21,22,23,25].

## 3. Chemical Structures and Biological Properties of the Actinomycetes Associated with Arthropods

### 3.1. Polyketides

Polyketides are a group of bioactive compounds found in nature with highly desirable chemical and bioactive properties. They possess a wide range of applications, from antibiotics, anticancer, and immunosuppressants to insecticides. Biochemically, these compounds are produced through a sequential decarboxylative Claisen-type condensation of acyl-CoA precursors, which is facilitated by enzymes known as polyketide synthases (PKSs). Depending on the function of several domains located in the PKSs with ketoreductase, dehydratase, and enoylreductase activities, the carbon skeleton of polyketides can be further reduced and modified. PKS structures vary significantly and are generally classified as one of three different types: type I, which are large multi-functional enzymes arranged into several modules; type II, which are multienzyme performing a single set of functions; and type III, which are iteratively acting condensing enzymes [32,33].

#### 3.1.1. Novel Compounds Isolated from the *Hymenoptera* Order

A high number of PKS-derive metabolites with antimicrobial activity have been isolated from this order including bees, wasps, and ants. Among these, the fungus-growing ants seem to receive special attention. One example of a well-studied defensive symbiosis is leaf-cutting ants of the *Attini* tribe, of the genera *Myrmicocrypta*, *Apterostigma*, *Acromyrmex*, and *Atta*, distributed by South America, Central America, and the southern USA. These ants live in a mutualistic beneficial relationship with *Basidiomycetes* fungi of the *Lepiotaceae* family forming a nest-house fungal garden. By collecting and cutting leaves, the ants provide a substrate for the fungi to cultivate. However, this beneficial relationship is threatened by the specialized fungal pathogen *Escovopsis*, which can overwhelm the gardens and eliminate the nest. As a defensive mechanism, the ants have developed behavioural adaptations, including symbiotic relationships with bacteria of the genus *Pseudonocardia*, which secretes antifungal compounds to protect the fungal cultivar. Nevertheless, in this highly competitive environment, another symbiosis has been established, mainly with bacteria belonging to the *Streptomyces* genus. This third partner produces many bioactive compounds that form part of the ant’s protective microbiome. In exchange, the symbiotic bacteria is provided with essential nutrients by the ant host [34].

**Figure 1:**
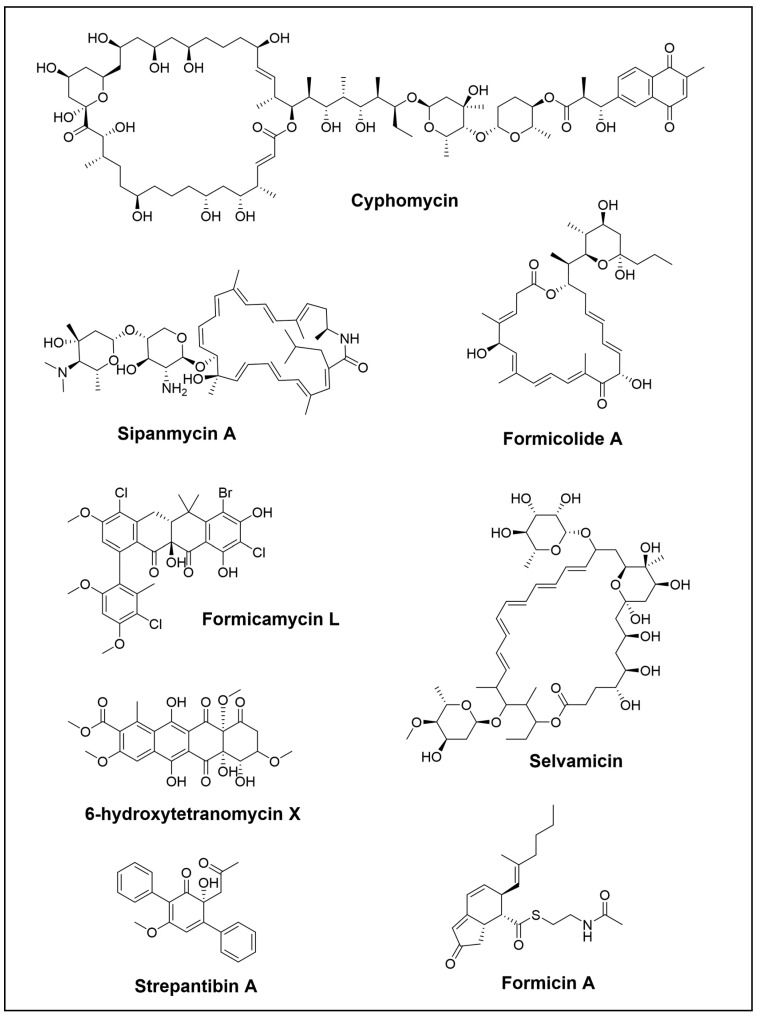
Novel polyketide compounds isolated from the *Hymenoptera* order.

Over the last few years, the investigation of fungus-growing ant species has guided the isolation and identification of numerous antimicrobial compounds, including some that are already known. Recently, Chevrette et al. have discovered cyphomycin (Figure 1) as a promising antimicrobial compound. It is a polyketide with a macrolide structure produced by a *Streptomyces* strain of the fungus-growing ants *Cyphomyrmex* sp. Cyphomycin has demonstrated strong in vitro activity against *Escovopsis* sp. and resistant human pathogens such as *Candida glabrata* 4720 (echinocandin resistance), *Aspergillus fumigatus* (triazole resistance), and *C. auris* B11211 (echinocandin, triazole, and amphotericin B resistance). The compound’s ability to combat fungi harbouring resistance mechanisms suggests that further studies are necessary to understand its mechanism of action and resistance. Additionally, cyphomycin has been shown to reduce infection levels in neutropenic mouse disseminated candidiasis models [21].

In another study of *Streptomyces* isolated from *Attini* fungus-growing ants, two novel macrolactams compounds, sipanmycin A and B, were discovered (Figure 1). In this case, the research conducted by Malmierca et al. illustrated an effective strategy for identifying new natural products that combined genome mining, a genetic approach, and chromatographic techniques. They screened seventy-one *Streptomyces* strains, isolated from the integument of ants, and searched for biosynthetic gene clusters (BGCs) that conducted to glycosylated compounds. Using a combination of PCR-base techniques, generation of mutants, and MS dereplication, two novel macrolactams were successfully discovered. Additionally, genetic and nutritional approaches were conducted to awaken the expression of silent clusters, which led to the identification of two novel members of the warkmycin family and the BGC of cervimycins [35]. Moreover, subsequent studies involved combinatorial biosynthesis by introducing plasmids harbouring genes for the biosynthesis of deoxysugars into a *Streptomyces* sipanmycin producer, which resulted in the production of six different sipanmycins with modified glycosylation patterns. In vitro cytotoxicity assays showed that the strongest activity was produced by the initial compound sipanmycin A. In the case of the derivatives that replaced D-spinose with an alternative deoxysugar, they exhibited significant IC50 values, indicating that the second deoxysugar plays a key role in the bioactivity of sipanmycins. Moreover, the novel derivates also had antibacterial activity against *Staphylococcus aureus* and *Micrococcus luteus* bacteria [36].

Two novel macrolides, formicolides A and B, have been discovered by An et al. (Figure 1). Formicolides were isolated from *Streptomyces* sp. BA01, which is a gut bacteria of *Formica yessensis* wood ant. Through genomic and bioinformatics analysis, the researchers were able to identify the type-I PKS pathway that employs a trans-acyltransferase system. Formicolides had antiangiogenic properties by suppressing tube formation in human umbilical vein endothelial cells. Moreover, they also promoted quinone reductase activity in murine Hepa-1c1c7 cells [37].

One unusual antifungal is selvamicin (Figure 1), a new macrocyclic that was discovered in a study of *Pseudonocardia* isolates from the basal fungus-growing ant genus *Apterostigma*. This compound shares a resemblance to two relevant antifungals, amphotericin B and nystatin A1 (both in the List of Essential Medicines by the World Health Organization), and to the food preservative and topical antifungal natamycin. What sets selvamicin apart from these compounds is a second sugar, a truncated macrocyclic core, and the lack of carboxylate and ammonium groups. Unlike amphotericin B and nystatin A1, which have high toxicity and minimal oral bioavailability, selvamicin exhibits better therapeutic properties and appears to operate via a distinct mechanism of action. Curiously, this compound was isolated from two bacterial isolates from two neighbouring ant nests. Genome analyses revealed that the selvamicin BGC is almost identical in both bacterial producers. Surprisingly, there was a difference in its location, with one situated on the bacterial chromosome and the other on a plasmid. This finding provides convincing evidence for horizontal gene transfer, highlighting the mobility of the BGC that underlies the variety and dissemination of defece secondary metabolites [38].

A new family of pentacyclic polyketide formicamycins and their biosynthetic intermediates, the fasamycins, have been discovered by McDonald et al. (Figure 1). These polycyclic polyketides are produced by *Streptomyces formicae* KY5 that were isolated from an African plan-ant denominate *Tetraponera penzigi* [39]. Two gene products are required for the transformation of fasamycins into formicamycin metabolites, and this process is carried out by a novel two-step ring expansion-ring contraction pathway [40]. Moreover, expression of the BGC, in which the repressor gene *forJ* has been deleted [41], into heterologous host strains *Saccharopolyspora erythraea* Δery yielded low levels of new glycosylated fasamycin. The formicamycins showed activity against vancomycin-resistant *Enterococcus faecium*, *Bacillus subtilis* methicillin-resistant, and *S. aureus* [8], while the glycosylated congeners lacked antibacterial activity [42].

Additionally, formicins A–C (Figure 1) were discovered from *Streptomyces* sp. associated with wood ants. Formicins A and B structures were elucidated as indenone thioesters bearing N-acetylcysteamine. Formicin A has been found to suppress the growth of human triple-negative breast cancer cells by controlling the liver kinase B1-mediated AMPK signalling pathway [43].

On the other hand, not only have new bioactive compounds been identified, but also new species. This is the case of Zakalyukina et al., who discovered a novel species of *Amycolatopsis* in adult ants *Camponotus vagus* using phenotypic, genetics, and phylogenetics approaches [44]. This new *Amycopatosis camponoty* sp. produced, among others, a valuable well-known metabolite tetracenomycin X and its new congener 6-hydroxytetracenomycin X (6-OH-TcmX) (Figure 1). Both have antimicrobial and cytotoxic activity, although 6-OH-TcmX has lower values and a comparable inhibition of in vitro protein synthesis. Their binding site for protein synthesis inhibition is located in a unique locus within the large ribosomal subunit. Its distinct mode of action makes it a promising option for additional structural diversification and pharmaceutical development [45]. Moreover, recently, biobricks toolbox for metabolic engineering of the tetracenomycin pathway has been developed and facilitated the production of biosynthetic analogues [46].

Aside from leafcutter ants, several interesting genera have been studied for the discovery of NPs in this order. One such example is the dauber wasp *Sceliphron madraspatanum* and its *Streptomyces* symbiont, which produced two new p-terphenyls, strepantibins A and B (Figure 1), along with the first representative of a naturally occurring bisphenyltropone, strepantibin C. It has been demonstrated that strepantibins C have antiproliferative effects on hepatoma carcinoma cells and inhibit the activity of hexokinase II (HK2) [47].

Another example is hamuracin C, a new bicyclic macrolide with noteworthy inhibitory activity against many human cancer cell lines, including SK-HEP-1, HCT116, SNU-638, A549, and MDA-MB-231. It was discovered through genomic and bioinformatic analysis of *Streptomyces* sp. MBP16, a gut bacterial strain of the wasp *Vespa crabro flavofasciata* [48].

#### 3.1.2. Novel Compounds Isolated from the *Blattodea* Order

**Figure 2:**
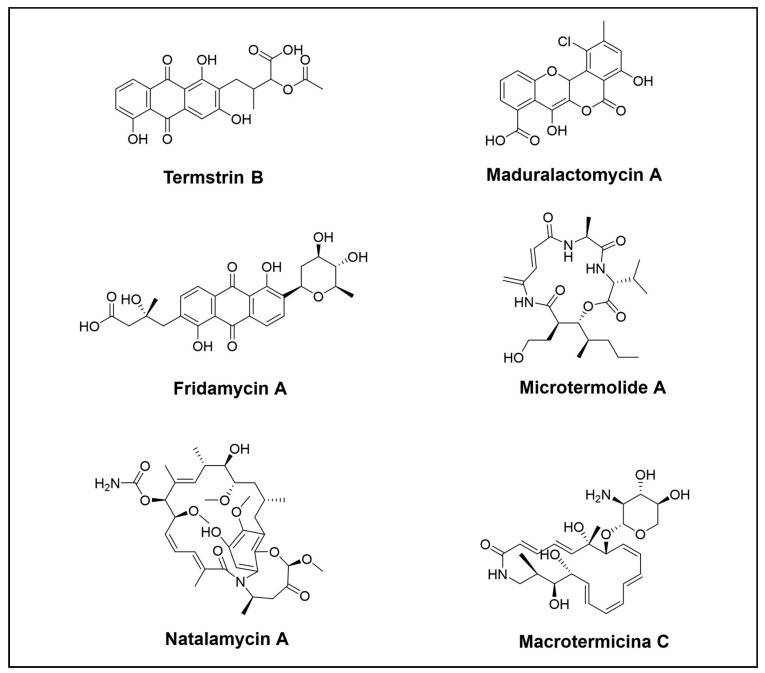
Novel polyketide compounds isolated from the *Blattodea* order.

It may seem surprising to know that the *Blattodea* order, which includes cockroaches and termites, is regarded as significant despite its relatively low species diversity compared to the *Hymenoptera* and *Coleoptera* orders. Termites, in particular, have been extensively studied and are known to possess the largest bacterial gut community among all insects [22]. Similar to fungus-growing ants, termite fungus-growing rely on a basidiomycete fungal cultivar as a primary food supply for their colony. In return, termites cultivate and clean the fungus garden and protect them from being infested by other species. Molecular docking and molecular dynamic simulation techniques have shown evidence that NPs derived from fungus-growing termites such as termstrin B (Figure 2) [49], fridamycin A (Figure 2) [50], maduralactomycin A (Figure 2) [51], and natalenamide C [52] have the potential to be inhibitors of *Acinetobacter baumannii*, one of the top six ESKAPE pathogens that caused multidrug-resistant hospital-acquired infections [53].

Termstrin A–D (Figure 2) are four anthraquinone derivatives isolated from termite-associated *Streptomyces* sp. BYF63. Termstrins A, B, and D exhibited antibacterial effects against *S. aureus*. Termstrin A also showed moderate cytotoxic activity against the tumour cell lines MGC-803 and A375 [49].

Fridamycin A (Figure 2) was isolated from *Actinomadura* sp. RB99 acquired from the surface of a termite worker. This compound has the potential to be a novel therapeutic candidate for management of type 2 diabetes [50]. Apart from this, the activation of the gene cluster of *Actinomadura* sp. RB29 leads to the discovery of the halogenated angucyclic maduralactomycins and spirocyclic actinospirols [51].

Other interesting chemical compounds from termites discovered by Beemelmanns et al. were microtermolides and natalamycen A (Figure 2). In their quest to uncover antifungal compounds, these researchers conducted bioassay-guided metabolomic analyses that led to the discovery of macrotermycins A–D (Figure 2). These newly glycosylated polyketide macrolactams were found in the actinomycete *Amycolatopsis* sp. M39. Interestingly, macrotermycins A and C exhibited selective antifungal activity against a fungal garden parasite, indicating their ecological role. They also showed antibacterial activity against *S. aureus* [54]. Subsequent genome mining in *Amycolatopsis* sp. M39 looking for gene clusters encoding macrolactams uncovered three previously unreported macrotermycin congeners [55].

#### 3.1.3. Novel Compounds Isolated from the *Lepidoptera* Order

**Figure 3:**
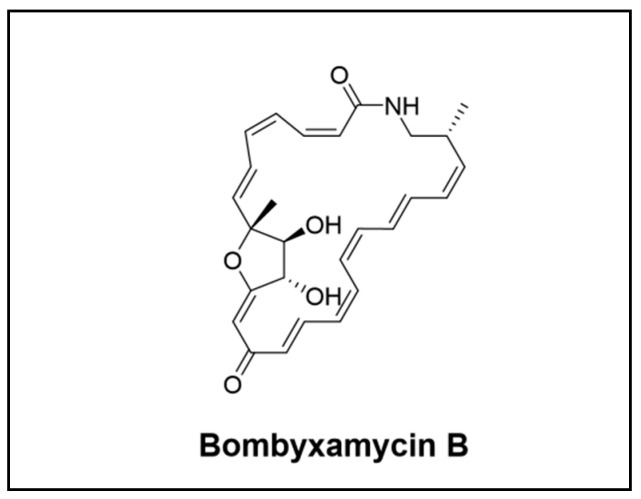
Novel polyketide compounds isolated from the *Lepidoptera* order.

The research on microbes associated with Lepidopteran insects has been limited. However, a noteworthy discovery has been the bombyxamycins A and B (Figure 3), which were found in a *Streptomyces* bacterium dwelling in the gut of silkworms. These compounds were identified as 26-membered cyclic lactams with polyene features and displayed antibacterial and antiproliferative properties. A further approach involving gene deletion experiments verified that the cytochrome P450 denominated BomK is responsible for the production of bombyxamycin B, which is unique due to its tetrahydrofuran in its macrocyclic ring [56].

### 3.2. Peptides

Peptide NPs have a variety of biological roles and an extensive spectrum of physico-chemical characteristics. In nature, complex peptide biosynthesis has diverged in two main ways: non-ribosomal peptide synthetases (NRPSs), and ribosomally synthesized and post-translationally modified peptides (RiPP). NRPSs are multi-enzyme complexes that biosynthesize non-ribosomal peptides (NRPS) in a modular line assembly and follow a similar chemical logic as PKSs for chain elongation. The chemical assortment of NRP lies in the integration of a broad range of amino and carboxylic acids, the existence of facultative enzyme domains module-encoded, online and offline tailoring reactions, and the fusion with other BGCs pathways. On the other hand, RiPP BGC encodes monofunctional enzymes and is smaller. Since RiPP’s precursors are limited to a series of 20 proteinogenic amino acids, they are converted to highly modified non-proteinogenic amino acids by an increasing number of RiPP modifying enzymes [57].

#### 3.2.1. Novel Compounds Isolated from the *Hymenoptera* Order

**Figure 4:**
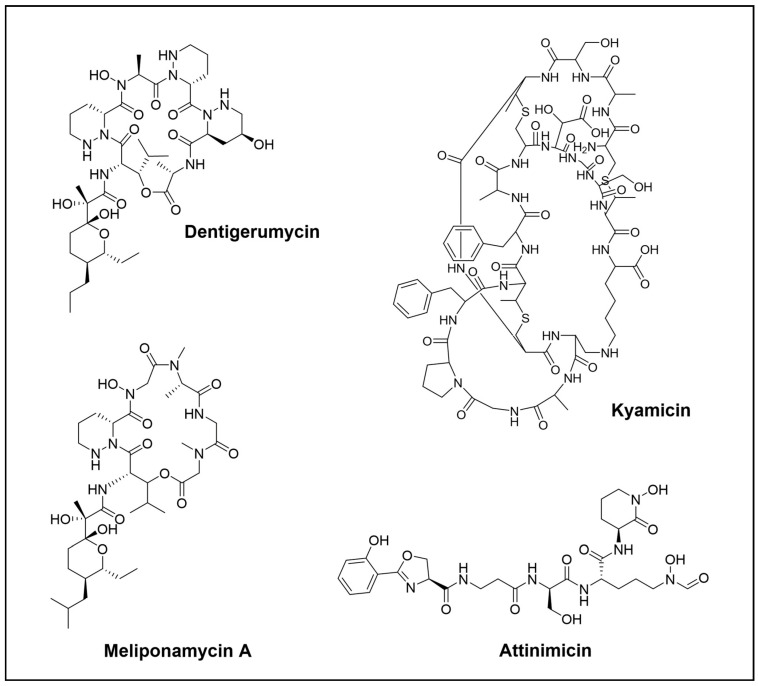
Novel peptide compounds isolated from the *Hymenoptera* order.

Dentigerumycin (Figure 4) is a cyclodepsipeptide that has an unconventional amino acid core skeleton including three piperazic acids, β-hydroxyleucine acids, N-hydroxylamine, and a polyketide-derived moiety with a pyran ring (Figure 4). This PKS-NRPS metabolite was isolated from the *Pseudonocardia* symbiont of the fungus-growing ant *Apterostigma dentigerum* and selectively inhibited the associated parasitic fungus *Escovopsis* sp. [58]. Curiously, in follow-up research, three new dentigerumycin analogues B-C were described, but in this case were identified in *Streptomyces* sp. M41 isolated forma a termite *Macrotermes natalensis* [59]. So far, a study based on replicating the environmental conditions in *Trachymyrmex* sp. ant nest led to the activation of cryptic dentigerumycin F, which is an antifungal chemical defence against *Escovopsis* sp. and *C. albicans* K1 [60].

A more interesting peptide is kyamicin (Figure 4), a new lanthipeptide antibiotic. Its cryptic BGC was identified with genome mining in a *Saccharopolyspora* species isolated from the obligate domatium-dwelling ant *Tetraponera penzigi* of the ant–plant *Vachelliawas*. To activate transcription, a SARP pathway-specific regulator was expressed under the control of a constitutive promoter. Finally, due to low production levels, heterologous expression was developed in *S. coelicolor* M1152 to enable the purification of the compound. The antibiotic antibacterial activity was studied and showed weak activity against *B. subtilis* EC1524 (MIC 128 µg/mL) [61].

Another different approach was made by Fukuda et al., who conducted a global study of Brazilian fungus-growing ant nests that led to the discovery of attinimicin (Figure 4), a powerful antifungal agent that is widely spread. This compound showed antifungal activity dependent on iron against specific environmental fungal parasites, but did not harm the fungal cultivar. It also had a strong in vivo activity in mouse infection models of *C. albicans*, similar to clinically used azole-containing antifungals. The geographic dissemination of the attinimicin BGC in Brazilian *Pseudonocardia* spp. indicated that attinimicin is the first specialized metabolite, known to date, from bacteria associated with ants with a wide geographic distribution [62].

On the other hand, bees, despite having a limited bacterial community, are an interesting group of insects responsible for the pollination of a broad range of crops and plants. The fact that honey has several antimicrobial properties has refuelled the interest in bee microbiota. As a result, two novel cyclic hexadepsipeptides, meliponamycin A and meliponamycin B (Figure 4) were discovered in *Streptomyces* sp. ICBG1318 isolated from *M. scutellaris* nurse bees. Both compounds showed strong activity against *S. aureus* and *Leishmania infantum*, as well as against the entomopathogen *Paenibacillus larva* [63].

#### 3.2.2. Novel Compounds Isolated from the *Blattodea* Order

**Figure 5:**
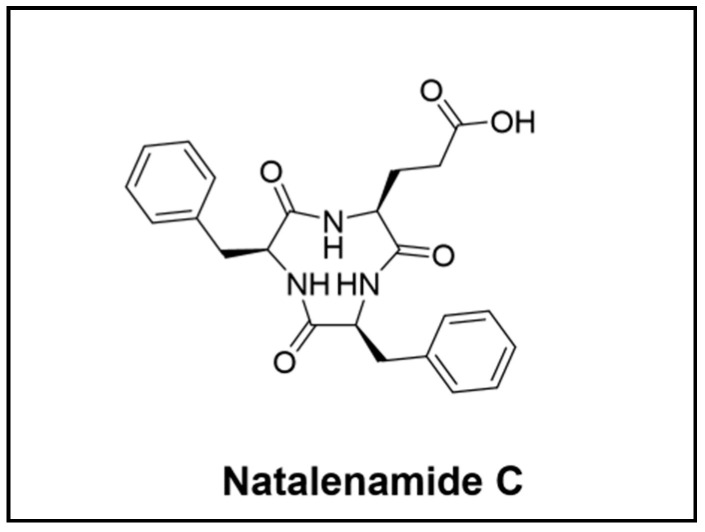
Novel peptide compounds isolated from the *Blattodea* order.

Lee and colleagues focus their research on *Actinomadura* sp. RB99 and its defensive functions in the fungus-farming termite *Macrotermes natalensis*. The study resulted in the discovery of natalenamides A–C, three new cyclic tripeptides (Figure 5), and the polyketide fridamycin (Figure 2). One of the tripeptides, natalenamide C, showed relevant inhibitory effects on 3-isobutyl-1-methylxanthine (IBMX)-induced melanin production [52]. The researchers also investigated if the siderophores produced by *Actinomadura* sp. RB99 in co-cultivates experiments could be responsible for the antimicrobial activity. These studies led to the identification of five madurastatin derivatives (A1, A2, E1, F, and G1), including a siderophore-metal complex [64].

#### 3.2.3. Novel Compounds Isolated from the *Coleoptera* Order

**Figure 6:**
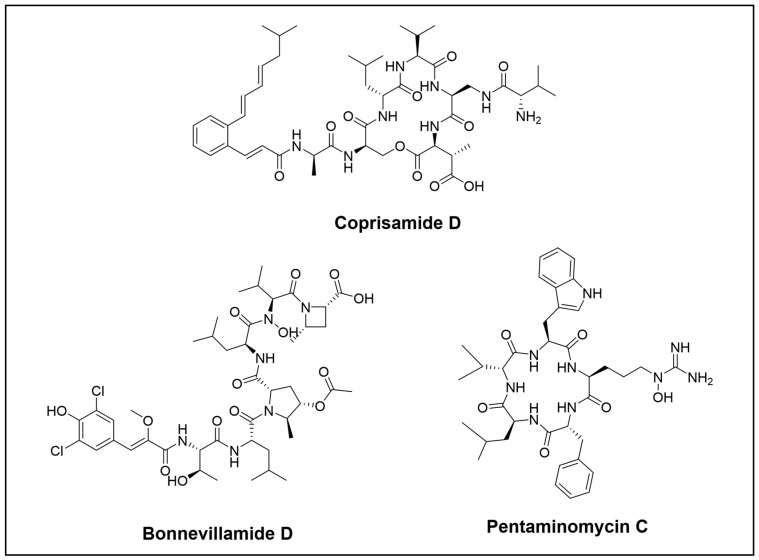
Novel peptide compounds isolated from the *Coleoptera* order.

In their research, Shin et al. discovered two new derivatives of coprisamides A and B, namely coprisamides C and D (Figure 6). These compounds are cyclic depsipeptides that contain a 2-alkenylcinnamic acid unit, as well as the unusual amino acids β-methylaspartic acid and 2,3-diaminopropanoic acid. They were isolated from *Micromonospora* sp. UTJ3, a gut bacterium which was found in the carrion beetle *Silpha perforate*. Coprisamides can induce quinone reductase (QR) activity and have a weak effect against the *Mycobacterium tuberculosis* mc2 6230 strains. In addition, Shin et al. reported two other new NRPS, bonnevillamides D and E (Figure 6), which were discovered in *Streptomyces* sp. UTZ13 that were found in the carrion beetle *Nicrophorus concolor*. These peptides have an anti-Alzheimer’s activity by reversing the fibril formation by inducing the monomerization of amyloid-β [56].

On the other hand, modifications in the culture conditions of a *Streptomyces* strain, which was isolated from the gut of the mealworm beetle, *Tenebrio molitor*, led to the production of cyclic pentapeptides, pentaminomycins C–E (Figure 6). Out of these, pentaminomycins C and D were found to be cytoprotective against oxidative stress in vitro and showed autophagy-inducing properties [65].

#### 3.2.4. Novel Compounds Isolated from Other Orders

**Figure 7:**
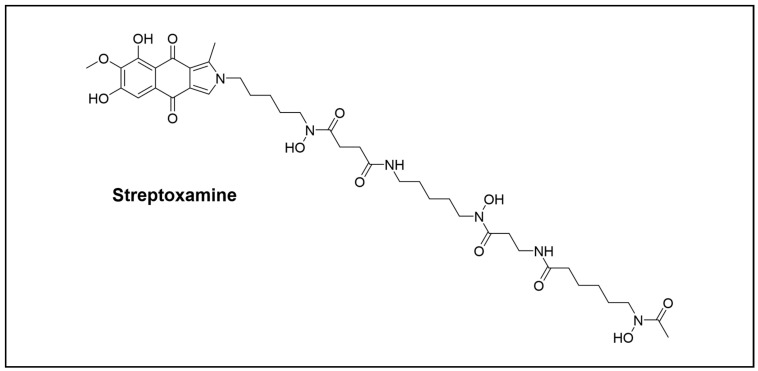
Novel peptide compounds isolated from the *Orhroptera* order.

The orders detailed so far are the most representative, but not the only ones that harbour NPs producers. For example, streptoxamine metabolite (Figure 7) discovered in *Streptomyces* sp. HKHCa2, which was isolated from *Oxya chinensis* belonging to the *Orthroptera* order. This compound is an unusual benzoisoindole deferoxamine hybrid, and displayed moderate antibacterial activity against *Mycobacterium smegmatis* and *S. aureus* [66].

On the other hand, some studies were conducted on crustaceans that belong to the Class *Malacostraca* in the order of *Decapoda*. For instance, *Streptomyces diastaticus* was isolated from a marine crustacean *Portunus*, and it has shown a promising antibiofilm activity against *C. albicans*. Further investigation for identification of the metabolite responsible for the antibiofilm activity is required [67]. In other research conducted by Axenov-Gribanov et al., *Streptomyces* and *Psudonocardia* species were isolated from the endemic Lake Baikal deepwater amphipods *Ommatogammarus albinus* and *Ommatogammarus flavus*. The strains demonstrated antifungal activity that could be related to the fungal resistance of amphipods [68].

### 3.3. Alkaloids

Alkaloids are a broad and structurally diverse group of NPs that have fascinated researchers due to their various biological activities, such as their analgesic, antibiotic, antibacterial, anti-virulence, antifungal, anticancer, anti-inflammatory, or anti-platelet properties. Their only unifying feature is the presence of a basic nitrogen atom. While most alkaloids only have one nitrogen atom, others can have as many as five. This nitrogen atom can occur in the form of a primary amine (RNH2), a secondary amine (R2NH), or a tertiary amine (R3N). Alkaloids can exist as monomers, or they can combine to produce trimers, tetramers, or dimers (bis-alkaloids). Usually, these oligomers are homo-oligomers, but they can also be hetero-oligomers. Alkaloids are not easily classified, and different guidelines may require concessions in borderline cases. They are usually classified based on their chemical features, biochemical pathways, or natural origin. According to the position of the N-atom in the main structural element can be divided into heterocyclic alkaloids, also known as typical alkaloids, which contain nitrogen in the heterocycle; non-heterocyclic alkaloids, also referred to as atypical alkaloids or proto-alkaloids, which contain nitrogen but not in the heterocyclic; polyamine alkaloids; and peptide alkaloids and the pseudo-alkaloids, which include terpene and steroid alkaloids [69].

#### 3.3.1. Novel Compounds Isolated from the *Blattodea* Order

**Figure 8:**
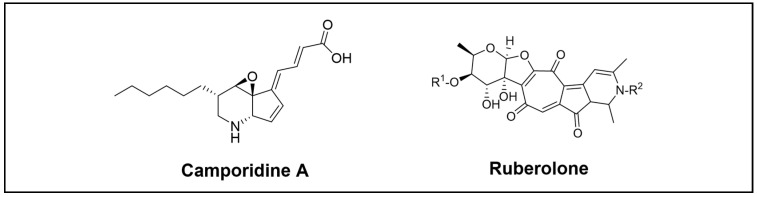
Novel alkaloid compounds isolated from the *Blattodea* order.

Camporidines A and B, two novel alkaloid compounds, were identified through chemical analyses of the gut bacteria *Streptomyces* sp. STA1 isolated from carpenter ants *Camponotus kiusiuensis* (Figure 8). These are new polyketide alkaloids with a piperidine-cyclopentene-epoxide 6/5/3 tricyclic system. By suppressing cell invasion, camporidine A demonstrated antimetastatic activity against the metastatic breast cancer cell line MDA-MB-231. It also displayed anti-inflammatory activity by inhibiting nitric oxide production induced by lipopolysaccharide. Additionally, the putative BGC of this class of polyketide alkaloids was discovered using bioinformatic analyses. The biosynthetic pathway was proposed as a modular type I PKS with an AT domain that accepts an atypical extender unit followed by post-PKS modification, including amination and oxidation along with spontaneous Schiff base formation. Moreover, the last post-PKS modification step proposed was the oxidation of the NH group on the piperidine ring of camporidin A to produce camporidin B, which could be catalysed by CamG, CamI, or CamT. Nevertheless, the precise mechanism for these final steps remains uncertain [70].

Beemelmanns et al. also reported the discovery of six new rubterolones A–F (Figure 8) produced by *Actinomadura* sp., 5-2, which was isolated from the gut of the termite *Macrotermes natalensis.* They are complex hybrid structures that feature a tropolone moiety, a fused cyclopentanone ring, an O, C-condensate sugar, and a high substitute pyridine or pyridinium inner salt moiety [71]. Follow-up analysis of the biosynthetic assembly line of rubterolones led to the identification of three highly reactive biosynthetic precursors, known as pre-rubterolones A–C. Additionally, a structurally diverse rubterolones compound library was generated. Furthermore, two new thiazoline-containing derivatives were afforded through fermentations in the presence of cysteine. Tests for biological activity showed that rubterolone and its derivates have an anti-inflammatory action [72].

#### 3.3.2. Novel Compounds Isolated from Other Orders

**Figure 9:**
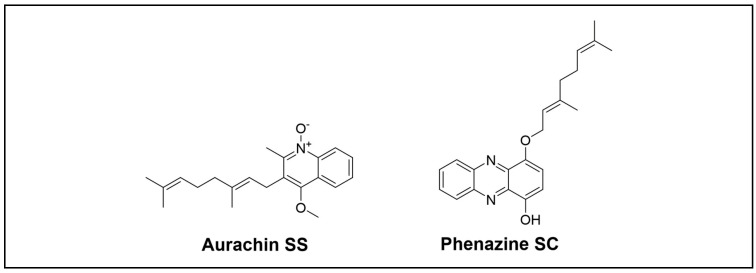
Novel alkaloid compounds isolated from the *Dermaptera* order.

Hao and colleagues researched earwigs that belong to the *Dermaptera* to explore NPs from microorganisms inhabiting unique environments. They reported a new type of antibiotic, aurachin SS (Figure 9) [73], along with three novel phenazines, SA to SC, from *Streptomyces* sp. NA04227 (Figure 9) [74]. Aurachin showed weak activity against Gram-positive bacteria. Phenazines SA to SC exhibited moderate AchE inhibitory activities, and phenazine SC displayed antimicrobial activities against *M. luteus*.

## 4. Conclusions and Prospects

Arthropods, through evolution, have been subjected to millions of years of continuous bioprospecting for active and defensive molecules. Additionally, pathogen pressure has resulted in the selection of strains that produce effective molecules, which can be tolerated by the host and actively inhibit specific external invasion, creating a coordinated microecological environment driven by chemical communication and chemical warfare [34]. This makes the investigation of actinomycetes associated with arthropods an incredible area for NPs discovery and drug development.

However, there are many gaps in knowledge, and to overcome those, we need more research data. For instance, while the key players are known, recent research indicates that they make up just a small fraction of the overall microbes present in any given ecological association [34]. In order to fully understand the symbiotic relationships and the functions that they perform, it is essential to comprehend the regulation of microbial specialized metabolites in their natural habitats.

Another drawback is that only a small number of in-depth investigations have been carried out on arthropod microbiomes, mainly in some orders of insects such as fungus-growing insects, whereas many other arthropods remain largely untargeted. Considering that arthropods constitute 50% of the animal biomass on Earth, it is clear that we have only scratched the surface of the potential of the microbial world. Therefore, expanding research to include arthropod hosts in extreme environments or associated with soil, plants, and organic debris could be a successful strategy for discovering new NPs.

An additional important issue is the difficulty of growing some strains in laboratory conditions. Currently, the most common method for discovering active strains is through traditional culture-dependent techniques. However, innovative approaches, such as the “ichip” method [73], have been developed to improve the efficiency of actinomycetes isolation.

To summarize, recent research has highlighted that we are only at the initial steps of the path leading to a complete understanding of the real diversity of the ecological relationships between actinomycetes and arthropods. This presents an opportunity to discover new bioactive compounds that could reinvigorate the industrial discovery pipelines. To unlock these hidden pathways, multidisciplinary approaches that integrate bioinformatics, genomics, ecology, and systems biology will be the key.

## Data Availability

The data presented in this study are available on request from the corresponding author.
